# Achieving the IA2030 Coverage and Equity Goals through a Renewed Focus on Urban Immunization

**DOI:** 10.3390/vaccines11040809

**Published:** 2023-04-06

**Authors:** Ibrahim Dadari, Rachel V. Belt, Ananya Iyengar, Arindam Ray, Iqbal Hossain, Daniel Ali, Niklas Danielsson, Samir V. Sodha, The Global Urban Immunization Working Group

**Affiliations:** 1 Coverage & Equity Unit, Immunization Section, PG-Health, UNICEF Headquarters, 3 UN Plaza, New York, NY 10017, USA; 2 College of Public Health, University of South Florida, Tampa, FL 33612, USA; 3 Nuffield Department of Women’s and Reproductive Health, University of Oxford, 75 George Street, Oxford OX1 2JD, UK; 4 Johns Hopkins Bloomberg School of Public Health, International Vaccine Access Center (IVAC), Baltimore, MD 21231, USA; 5 New Vaccines and Immunization Systems, Bill and Melinda Gates Foundation, New Delhi 110067, India; 6 John Snow, Inc., 2733 Crystal Drive, Arlington, VA 22202, USA; 7 Department of Immunization Vaccines and Biologicals, WHO Headquarters, Avenue Appia 20, 1211 Geneva, Switzerland

**Keywords:** zero-dose, urban immunization, catch-up and recovery, un-immunized, under-immunization, routine immunization

## Abstract

The 2021 WHO and UNICEF Estimates of National Immunization Coverage (WUENIC) reported approximately 25 million under-vaccinated children in 2021, out of which 18 million were zero-dose children who did not receive even the first dose of a diphtheria-tetanus-pertussis-(DPT) containing vaccine. The number of zero-dose children increased by six million between 2019, the pre-pandemic year, and 2021. A total of 20 countries with the highest number of zero-dose children and home to over 75% of these children in 2021 were prioritized for this review. Several of these countries have substantial urbanization with accompanying challenges. This review paper summarizes routine immunization backsliding following the COVID-19 pandemic and predictors of coverage and identifies pro-equity strategies in urban and peri-urban settings through a systematic search of the published literature. Two databases, PubMed and Web of Science, were exhaustively searched using search terms and synonyms, resulting in 608 identified peer-reviewed papers. Based on the inclusion criteria, 15 papers were included in the final review. The inclusion criteria included papers published between March 2020 and January 2023 and references to urban settings and COVID-19 in the papers. Several studies clearly documented a backsliding of coverage in urban and peri-urban settings, with some predictors or challenges to optimum coverage as well as some pro-equity strategies deployed or recommended in these studies. This emphasizes the need to focus on context-specific routine immunization catch-up and recovery strategies to suit the peculiarities of urban areas to get countries back on track toward achieving the targets of the IA2030. While more evidence is needed around the impact of the pandemic in urban areas, utilizing tools and platforms created to support advancing the equity agenda is pivotal. We posit that a renewed focus on urban immunization is critical if we are to achieve the IA2030 targets.

## 1. Background

The most dramatic declines in routine immunization coverage in the last 30 years occurred in part due to the COVID-19 pandemic. There were approximately 25 million un-vaccinated or under-vaccinated children in 2021, out of which 18 million were zero-dose, based on the proxy indicator of the number of children who did not receive the first dose of a diphtheria-tetanus-pertussis-(DPT) containing vaccine [1]. This drop in coverage has resulted in an increase in the number of zero-dose children globally, from 13 million in 2019 to 18 million in 2021 (approximately 40% more zero-dose children) [2].

A greater focus on strategies to reach un- and under-vaccinated children in urban, rural, and conflict settings is encouraged by the Equity Reference Group on Immunization (ERG), as these populations are facing acute inequities [1]. Due to rapid global urbanization, the world’s population is at least 56% urbanized as of 2020 and expected to reach 70% by 2050 [2]. This fast urbanization is not devoid of complications, especially in low- and middle-income countries (LMICs) where the numbers of large informal settlements and urban-poor populations are growing, resulting in huge inequalities in access to basic primary healthcare services, including immunization [3], as such a shift in the distribution of zero-dose and under-vaccinated children towards urban environments is envisaged [3].

The COVID-19 pandemic was declared a public health emergency of international concern in early 2020, and as of February 2023, over 755 million cases and 6.8 million deaths were reported [4]. Routine immunization faced backsliding of coverage due to the pandemic with more than 5 million additional zero-dose and under-vaccinated children added to the 2019 baseline globally. Disruptions to routine immunization occurred on a global scale, with low- and middle-income countries being the hardest hit, due to less resilient routine immunization systems. National lockdowns and restrictions of movements interrupted the provision of medical services and resulted in large swaths of the population either not having access to services or feeling nervous to access these services due to fear of COVID-19 transmission. Pakistan and India are said to have reported major drops in immunization coverage, with Pakistan reporting a substantial coverage decline in all childhood immunization services during the pandemic lockdown [5,6]. Due to data limitations, the extent of decrease in service coverage in some African countries has not been fully elucidated. However, the trend appears to indicate that countries with lower pre-pandemic immunization coverage trends saw more significant drops in performance than countries with higher immunization rates [5]. The routine immunization coverage trajectories as of the end of 2021 indicate some level of recovery but still show warning signs that without concerted efforts to strengthen immunization systems, gaps in coverage will persist [5,7]

A 2022 estimation of the number and distribution of zero-dose and under-immunized children within remote-rural, urban, and conflict-affected locations from 99 LMICs, showed approximately 30% of zero-dose children are in urban and peri-urban areas, compared with remote-rural areas which have about 11% [8]. A substantial proportion of these zero-dose or under-vaccinated children in urban settings are in slums and informal settlements, and these numbers are projected to increase with the current trends of rapid urbanization. These urban settings have their own context-specific challenges needing distinct approaches, including a larger proportion of transient or migrant populations, unclear catchment areas for health facilities, lack of appropriately disaggregated data, disenfranchised communities, increase in informal settlements, insecurity, and satisfying multiple stakeholders including private providers [3].

A major indicator of the Immunization Agenda 2030 (IA2030) [9,10] is a reduction in the number of zero-dose children by 50% by 2030, but this indicator is off-track in large part because of routine immunization backsliding. A total of 20 high-burden countries (Appendix A.1) are home to over 75% of the world’s zero-dose children as of 2021 [11]. These countries are largely urbanized, with a considerable proportion of their zero-dose children localized in urban settings, as exemplified by 4 of these 20 focus countries accounting for over 50% of the global population of zero-dose children. These countries include Brazil, Mexico, Indonesia, and Nigeria, with 87%, 81%, 57%, and 53% urbanization, respectively [12]. Many countries have developed urban-specific immunization strategies or highlighted urban approaches in their comprehensive multi-year plan for immunization (cMYP) or national immunization strategies (NIS). Some of these countries attribute their quick recovery from the COVID-19 pandemic to an active urban approach being implemented in their countries.

To address issues of urban immunization, the Urban Immunization Working Group (UIWG) was established. The UIWG is an extra-organizational group comprising policy, program, and academic experts, representing their respective organizations, created to inform global, regional, and national/subnational discussions on immunization equity in urban areas [13]. The group meets virtually or in person 2–4 times every year, facilitated by UNICEF, and has broad membership across key immunization stakeholders. In addition to fostering collaboration and alignment among partners working to improve immunization coverage in urban settings, the urban immunization toolkit, which is now being used by many, is one of the group’s products. The urban diagnostic research across several countries was also supported by the working group. The group continually discussed emerging issues and defined a trajectory for future engagement to strengthen the focus on immunization in urban settings.

To facilitate getting countries back on track toward achieving the IA2030 goals, this paper highlights the importance of a renewed focus on addressing the idiosyncrasies of immunization in urban settings through a review of existing data and information on (i) the estimated magnitude of coverage backsliding in urban and peri-urban settings in a select set of countries, (ii) key issues affecting routine immunization in urban and peri-urban settings, and (iii) effective pro-equity strategies for routine immunization recovery in urban settings. The outcome of this study will be shared with global and national immunization stakeholders to provide evidence on effective strategies to vaccinate children in urban-poor communities.

## 2. Methods

We conducted a systematic search of the published literature to identify relevant information and data on backsliding of immunization coverage in urban settings during the COVID-19 pandemic, the predictors and challenges of routine immunization and pro-equity strategies relevant to urban and peri-urban settings. Priority was given to the 20 high-burden zero-dose countries, per the 2021 WHO and UNICEF Estimates of National Immunization Coverage (WUENIC). This study has the following research questions prioritized for the review:

1. To what extent did COVID-19 pandemic interrupt routine immunization performance and other related services in urban and peri-urban settings in focus countries?
2. What were the predictors of decline or backsliding in immunization coverage in these settings?
3. What was done to recover immunization coverage?

The literature reviewed were from 2 databases, namely, PubMed and Web of Science, due to their broad coverage of health sciences, and inclusive of published literature in the 20 high-burden zero-dose countries for the period between March 2020 and January 2023, to cover the period of COVID-19 pandemic. Search terms used were “immunization coverage” and “urban” and were limited to twenty countries and the period of March 2020 to January 2023. The paper types include ecological studies, cross sectional, interventional, and pre- and post-studies. The full search strategy is available in Appendix A.2.

**Figure 1:**
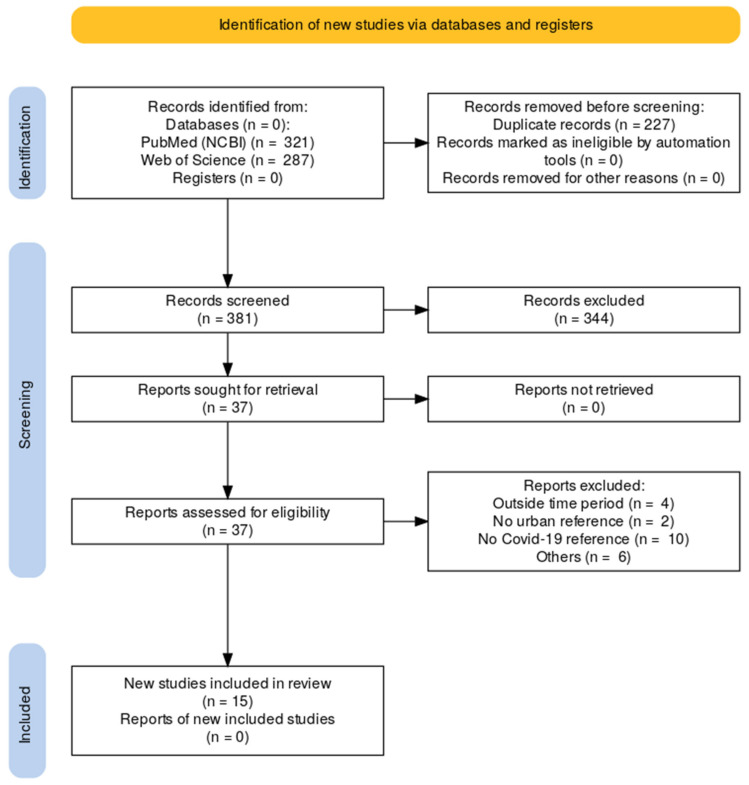
PRISMA Flow chart for the review of papers.

A total of 608 articles were identified from both databases (PubMed = 321 and Web of Science = 287), which were imported into Rayyan (rayyan.ai). Screening of records detected and removed 227 duplicates. The title and abstract screening of 318 articles based on our inclusion criteria was conducted by 2 authors, ID and RB. The blind decisions by both authors for inclusion were 90% aligned. The reviewers reviewed the misaligned articles and agreed on the final decision according to the criteria in Table 1. Thirty-seven articles were identified for inclusion after the title and abstract review. The remaining articles were screened and excluded if the papers fell outside the designated time period, did not contain references to urban populations or areas, and did not contain references to COVID-19. A flow chart for the literature review is included in Figure 1. A final 15 articles were included in the review, analyzed, and presented in this study.

## 3. Results

The findings from this study are organized according to the study objectives, which are (i) the backsliding of immunization in urban, (ii) immunization challenges and predictors in urban and peri-urban settings, and (iii) identified pro-equity strategies. 

### 3.1. Backsliding of Immunization in Urban

Some papers provided an estimation of the magnitude of coverage backsliding in country contexts that are mostly urbanized or in context of urban and peri-urban settings. A nationwide ecological study in Brazil looked at the coverage figures of yellow fever vaccination before and during the COVID-19 pandemic. This study was conducted between April 2019 and March 2021 and found a 48.55% decline in the median yellow fever vaccination doses administered nationwide 1 year after pandemic control measures were instituted (April 2020–March 2021) compared to the pre-pandemic period (April 2019–March 2020) [14]. Some of the states with substantial decline rates included Paraná (49.97%), Sao Paulo (43.25%), and regions such as the North (34.71%), Midwest (21.72%), South (63.50%), and Southeast (34.42%). Brazil is a largely urbanized country with urbanization between 65% and 90% across the states. 

A 2022 multi-city phenomenological qualitative study in India by Sahoo et al. documented the experiences of urban slum-dwelling women with maternal and child health services during the COVID-19 pandemic [15]. This study was conducted in four cities (one city with a dense slum population per state) across four states: Bhubaneswar Municipal Corporation (Bubaneswal, Odisha, India), Rishikesh Municipal Corporation (Rishikesh, Uttarakhand), Bhilai Municipal Corporation (Chetis Gerbilee, Chhattisgarh, India), and North Lakhimpur Municipal Board (North Lakhimpur, Assam, India). All participants reported getting their children vaccinated during the pandemic with little or no backsliding reported. 

Manzoor and colleagues conducted a cross-sectional study to document the impact of the COVID-19 pandemic on routine childhood immunization in Mirpur, Azad Kashmir, Pakistan [16]. The study found that the COVID-19 pandemic had a major impact on the timing of routine immunization for children in Pakistan, where about 80% of caregivers had scheduled vaccinations for their children, 18% had delayed vaccination schedules, while 2% missed vaccination during the COVID-19 pandemic. The fear of contracting the COVID-19 virus was a key factor that resulted in delaying vaccination mentioned by about 65% of participants, and 40% reported that home is the preferred location to get their children vaccinated. 

A comparative cross-sectional study in Wolaita of southwest Ethiopia documented the disparities in full immunization coverage among urban and rural children aged 12–23 months [17]. The study found that knowledge of and attitude towards immunization and fear of COVID-19 at the health facility and place of delivery were predictor variables for full vaccination coverage and that urban children had a higher full vaccination than rural children by a 15% point estimate. 

A natural experiment, which assessed the impact of the COVID-19 pandemic on coverage of Reproductive, Maternal, and Newborn Health interventions in Ethiopia at the early stages of the pandemic, showed a significant reduction in coverage of BCG vaccination and chlorohexidine use in urban areas amongst the cohort impacted by COVID-19 outbreaks with little or no significant reductions in women seeking either preventative or curative health services [18]. 

A descriptive and retrospective cross-sectional study conducted in Yaoundé, Cameroon, showed a decline in the number of pediatric consultations by 52% in April and by 34% in May 2020, compared with rates during the same periods in 2019 (*p* < 0.01), following the partial confinement recommended by the government [19]. The demand for BCG vaccines, third dose of DPT, polio, and MMR in children, as well as tetanus vaccines in childbearing women showed a decline [19].

A 51% decline in the daily average total number of vaccinations administered during lockdown compared to the baseline was found throughout the Sindh province in Pakistan from a pre- and post-study analyzing provincial electronic immunization data; the paper examined the impact of the COVID-19 pandemic response on the uptake of routine immunizations with the highest decline seen in BCG vaccines at fixed sites [6]. It was also noted that slum union councils had a slightly larger decrease in immunization coverage than non-slum urban areas (53.8% vs. 51.3%). Some of the predictors of getting vaccinated included children born at health facilities and children of mothers with higher education levels.

Based on a study that analyzed routine statistics and a national household survey in Brazil, a decline of about 20% in vaccines administered to children aged 2 months or older was seen during March and April 2020, when social distancing was at the highest level compared to January and February of the same year [20]. This study also showed that children from poor households and the least developed regions of the country were most affected compared to other children.

### 3.2. Immunization Challenges and Predictors in Urban and Peri-Urban

A study in four cities of India identified some issues such as no first-time registration for childhood vaccination in Uttarakhand and children not receiving an associated package of health services such as child weighing in Chhattisgarh [15]. Despite the positive outlook for childhood vaccination, other maternal and child health services, including treatment of the sick child, and postnatal care suffered due to the COVID-19 pandemic. 

A population-based longitudinal study was conducted by Meckonnen and colleagues in Kersa [21]. Through face-to-face interviews, data was collected from caregivers of over 14,000 children. Harar city children had a 45% coverage rate for full vaccination, while conversely, other towns classified as semi-urban showed the lowest level of full vaccination coverage. Overall, 39% of children were found to be fully vaccinated. Being in a semi-urban residence, older maternal age, rural residence, maternal education, and unemployment were associated with not being vaccinated. Some of the barriers responsible for low routine immunization coverage in urban settings included poor defaulter tracking mechanisms for urban children, unfriendly immunization service delivery in urban public health facilities due to overstretched human resources, lack of effective strategies to reach the most vulnerable and marginalized urban communities with vaccines, and private service provider barriers, to name a few.

A community-based cross-sectional mixed-method study conducted in Toke Kutaye district, central Ethiopia, assessed vaccination timeliness and associated factors among children [22] and found an overall timeliness of childhood vaccination of only 23.9 percent among children aged 12 to 23 months, making other children who did not receive timely vaccines vulnerable. Urban residence, participation of pregnant women in conferences, and institutional delivery are among the independent predictors associated with the timeliness of childhood vaccination.

A descriptive cross-sectional survey of adolescent girls’ parents conducted in two urban and two rural secondary schools in Lagos, Nigeria, documented parental acceptance of human papillomavirus vaccination for adolescent girls [23]. Urban residence among other factors such as tertiary level of education in the mother, skilled occupation of both parents, and knowledge of HPV were all positively associated with getting vaccinated with HPV vaccines.

Tadesse and colleagues explored associated factors related to second-dose measles vaccination among under-five children in urban areas of North Shewa Zone, Oromia, Ethiopia, using a community-based cross-sectional study [24]. The study found a low (42.5%) level of second-dose measles vaccination (MCV2) among children in urban areas of the study area. Some of the predictors of MCV2 uptake included maternal age, average time mothers had been waiting for vaccination at the health facility, awareness about vaccine-preventable diseases, awareness around recommended age for the last MCV vaccine in the series, and knowledge of the recommended number of MCV doses. A lack of information was the major reason for children not getting the MCV2 vaccination.

Findings from a study in Cavite, the Philippines, which assessed hesitancy towards vaccines among caregivers using in-depth interviews, documented that among the reasons for delay or refusal of childhood vaccinations, fear of side effects emerged as the most salient concern, exacerbated by previous negative experiences (including trauma) from a dengue vaccine controversy in 2017. Respondents also highlighted religious, cultural, and health system factors, including appointment scheduling and waiting times as predictors of childhood vaccination [25].

### 3.3. Identified Pro-Equity Strategies

Some strategies to ensure full vaccination during the pandemic were also highlighted in some of the papers reviewed. In one such paper, parents from multiple states in India chose to use private hospitals for child immunization due to the fear of themselves or their kids getting infected with the COVID-19 virus [15]. Some of the facilities in these cities considered shortening the waiting time for routine childhood vaccination service delivery, while others changed their vaccination timing schedule to reduce the spread of infection during the COVID-19 pandemic.

Meckonnen and colleagues conducted a phenomenological qualitative study to document strategies to revitalize immunization service provision in urban settings of the cities of Addis Ababa, Dire Dawa, and Mekele in Ethiopia [26]. Their study found that the immunization service provision strategies existing during the study period in urban settings were not adequate to reach all children and are mostly static (fixed) sessions. Some of the proposed strategies included expanding routine immunization service access to marginalized populations through outreach services, strengthening the public–private partnership, engaging the private health facilities for vaccination services, and integrating technological innovations (such as digitalization of the EPI program and application of mHealth reminders) to facilitate inter-facility linkage. 

Balogun and colleagues conducted a pre- and post-interventional study in seven urban slum communities in Ibadan, Nigeria, in 2020–2021 to document the effect of intensive training in improving older women’s knowledge and support for infant vaccination in Nigerian urban slums [27]. Identified older women received training through participatory learning methods over an 8-month period with a manual and short video on the importance of immunization timeliness and completion, how vaccines work, and how to be advocates and supporters of infant vaccination. It was shown that participatory learning improved the knowledge of these older women who provide support and supervision for childcare in urban slums about vaccination and how to better support infant vaccination.

The study from Oromia (Tadesse et al.) also recommended some strategies for increasing the uptake of MCV2, including shortening the waiting time for vaccination at the health facility to within half an hour, intensifying awareness for parents and caregivers, and paying particular attention to mothers who are older than 36 years of age [24].

## 4. Discussion

With the rapid globalization seen in many countries, it is imperative to proactively identify and address urban-specific challenges to routine immunization, including mapping and reaching zero-dose children and missed communities. This renewed focus will assist efforts toward extending the reach of vaccines in urban settings and contribute to achieving the IA2030 targets. Already, it is estimated that about 30% of zero-dose children live in urban and peri-urban areas [8], and these numbers could grow rapidly without sufficient focus and proactive interventions. A key step, which this paper has taken, is to review evidence on the backsliding of coverage and routine immunization performance in urban settings and select focus countries harboring more than 75% of zero-dose children globally, documenting predictors of coverage and pro-equity strategies. Findings from this review show evidence of backsliding and disruption across the globe in urban and peri-urban contexts; major urbanized countries from around the world, such as Brazil, Pakistan, Ethiopia, India, and Cameroon, show various levels of immunization performance disruption in multiple urban contexts [6,15,18,19,28]. The disruptions to immunization affected multiple vaccines in each country’s routine immunization schedule, including BCG, yellow fever (YFV), and DPT. Many challenges varied across the different global contexts, including no first-time immunization registration for children, poor tracking mechanisms for urban children, unfriendly delivery, and lack of effective urban-immunization-specific strategies; similarly, varied determinants of higher immunization coverage included institutional delivery of children, higher maternal education, lower maternal age, and positive knowledge and attitudes around immunization. Of the identified pro-equity strategies, shortening waiting time for service delivery, improved outreach services, and promotion of maternal and female figure education were associated with higher levels of immunization coverage. The predictors of childhood vaccination in urban settings, as well as noted pro-equity strategies, as documented in these studies, are consistent with what has been documented pre-pandemic [29,30,31].

These results suggest several potential areas for effective interventions to accelerate inroads into urban immunization. The identified pro-equity strategies above link to challenges on immunization and higher immunization coverage levels, and while there is a lot of diversity in backsliding issues, successful pro-equity strategies were also tailored to specific contexts, which emphasizes the need to contextualize interventions to address specific idiosyncrasies in each context [32]. Some results were contradictory to the idea that urbanized populations can be associated with poorer vaccination coverage, but most of the studies supported this idea. Additionally, this is not surprising as the world population transitions to become more urbanized [12], especially with the limited studies focusing on the peculiarities of essential vaccination in urban and peri-urban contexts.

This review contributes to a clearer understanding of the post-pandemic landscape of urban immunization in low- and middle-income countries, including challenges, immunization coverage determinants, and most importantly, pro-equity strategies. These pro-equity strategies are essential to ensuring that vaccines reach under-served populations and missed communities, and in examining these strategies more closely, we can better generate a starting point for a roadmap to longer-term urban immunization information. These are consistent with earlier documentation of pro-equity strategies in Gavi-supported countries [33]. 

Contextualized pro-equity urban immunization interventions will result in faster advances toward the IA2030 targets, and work to halt and slow down current backsliding and fragility in immunization systems. Additionally, many of the results point to the room for multisectoral and integrated interventions, as many of the determinants related to low immunization coverage point to potential interventions on gender barriers, education barriers, and systems strengthening [32,34,35]. Tools such as the urban immunization toolkit are being used by several countries to complement existing immunization guidelines by tailoring immunization planning, implementing, and monitoring approaches to meet challenging contexts in urban areas, especially in slum environments; many such tools are available to support these efforts globally [35]. 

There are some limitations to the data reviewed, including its quantity, generalizability, and whether urban-specific immunization challenges and contexts can be differentiated from generalized immunization challenges and contexts. There were a limited number of studies examining the post-pandemic urban immunization landscape, and the evidence was limited to a relatively small number of countries. Studies that concentrated on the slums were included alongside other peri-urban and urban settings across many different countries, and due to the evidence being contextualized, it may not be possible to generalize these findings. Additionally, many of the studies reviewed were only conducted in urban settings, so evidence of uniquely urban-related challenges is somewhat limited, as these challenges could apply in other settings. Furthermore, the definitions of urban and peri-urban used across the studies may have varied, which could have affected the findings and interpretation.

Because there is a relative dearth of information about urban immunization occurring after the acute phase of COVID-19, and much more information about the acceleration of global immunization and backsliding, it is essential that more studies be conducted on this intersection, as it is crucial to have more disaggregated data around urban backsliding, immunization performance evidence, and pro-equity strategies. Issues specific to the urban context need to be differentiated from generalized data in order to contextualize and prioritize the necessary correlated interventions and pro-equity strategies. 

### 4.1. Policy Suggestions

(a) Multisectoral innovations: many of the drivers of immunization performance highlighted from this and other reviews transcend ‘traditional’ determinants of immunization performance (such as vaccine supply or trained community health worker availability). Addressing other social issues such as maternal education, access to water, sanitation, and hygiene (WASH) services, and (maternal and child) education is pivotal in improving immunization performance in urban settings [32], especially governance in the immunization space around urban and should be inclusive of these sectors for more robust programming.
(b) Strengthen partnerships: leveraging the comparative strengths and expertise of partners across immunization, health, and other sectors such as planning and sanitation will be valuable. For instance, the Mission Indradhanush (MI) in India, which worked across sectors, recorded significant gains in immunization coverage in both urban and rural settings [36]. Considering the diversity of non-government actors in urban areas, a purposeful policy shift to further incorporate private service providers and non-governmental organizations into immunization service delivery is needed.
(c) Monitoring and Data: The typical immunization data built upon traditional subnational administrative boundaries may not suffice to effectively monitor, measure, and track children in urban settings [13,35]. The urban population is fast growing and in motion most of the time. Modern innovations in digital registries and data tracking systems could provide answers on how to effectively track these inherently transient and migratory populations or populations that may not have access to the traditional identifications used in accessing government health services. Periodic routine micro census and the use of geographic information system (GIS) enable data to have shown great promise [8,37]. More enhanced disaggregation of immunization data by urban in routine data systems, such as the WHO and UNICEF joint reporting form (JRF), as well as in coverage surveys will support monitoring and tracking of immunization services in urban areas.

The above and other context-specific policy adjustments should be considered to make improvements and tend toward achieving the IA2030 goals.

### 4.2. Future Studies

The limited studies available and reviewed in this review point to the need for more evidence generation and documentation, including documenting and sharing tested innovations within the urban context. These are not only essential but also have the potential to be incredibly innovative and informative. Many countries included in the search have not performed or reported studies on immunization performance in urban contexts during or after the pandemic. Future studies could consider examining the efficacy and effectiveness of innovations, urban settlement micro plans and micro tracking mechanisms, GIS mapping, the intertwining of conflicts, displacements and urban immunization, digital registry systems, and how urban immunization plans can be combined with other social sector innovations.

## 5. Limitations

The literature search was limited to the top 20 zero-dose countries based on 2021 WUENIC data; these, however, may not be fully representative of other countries. Only 15 papers were eligible after screening, and some of the 20 countries were not represented at the conclusion of the literature review, indicating a limitation of existing literature on the subject. Grey literature and other non-published reports were not included in this review. Some of the studies did not fully elucidate the nuances of challenges faced in urban or peri-urban settings.

## 6. Conclusions

The findings presented clearly demonstrate the evidence of a decline in routine immunization coverage in urban and peri-urban settings; as such, the need to target and focus context-specific catch-up and recovery strategies to bring back routine immunization performance on track. However, they also elucidate the need to further explore and examine determinants of low immunization coverage in urbanized areas, particularly in low- and middle-income countries. There are relatively few papers that estimate the magnitude of backsliding caused by COVID-19 in urban and peri-urban settings, in these priority countries, as well as a dearth of papers focusing on key issues affecting routine immunization in these settings and effective pro-equity strategies to address those keys issues in immunization recovery. While the COVID-19 pandemic has only been affecting immunization coverage in these areas for almost three years, the backsliding caused by this international disruption has proved detrimental, particularly toward reaching the goals set by IA2030.

Over the many different contexts explored through the review, different factors have been shown to affect immunization coverage in urban and peri-urban areas. It is essential that each context is specifically examined for services to be designed and tailored to the communities affected by the lack of access to these services. Innovations in interventions will be needed to build a better pro-equitable system of immunization for these areas. Rapid global urbanization makes addressing urban immunization challenges an essential and immediate priority in order to keep up with the demands placed on global immunization systems in the 21st century. 

## Figures and Tables

**Table 1:** Inclusion and exclusion criteria for literature search.

Criteria	Inclusion	Exclusion
Language	English, Spanish, French	Other languages
Dates	March 2020–January 2023	Before March 2020, unless analysis reviews general immunization trends pre-March 2020 to post-March 2020
Database	PubMed (NCBI), Web of Science (Clarivate)	Other databases
Vaccines	All routine vaccines administered to children up to age of 18 years.	Exclude COVID vaccines and vaccines administered to adults >18 years.
Topic	COVID-19 impact on routine immunization and recovery	Articles which do not focus on the COVID-19 impact on routine immunization and recovery
Geographic Location (Countries)	India, Nigeria, Indonesia, Ethiopia, Philippines, Democratic Republic of the Congo, Brazil, Pakistan, Angola, Myanmar, United Republic of Tanzania, Mozambique, Afghanistan, Somalia, Mexico, Madagascar, Cameroon, Democratic People’s Republic of Korea, Chad, Vietnam	All other countries
Geographic Location (Urban)	Urban and peri-urban, cities, Urban vs. rural analysis	Rural

## Data Availability

All data and publications used are referenced and available in the related databases.

## Appendix A

### Appendix A.1. Immunization Coverage Performance for the 20 Highest Burden Zero-Dose Countries

**Rank**	**Country**	**DTP1 (%)**	**DTP3 (%)**	**MCV1 (%)**	**Number Zero-Dose Children**
1	India	88	85	89	2,711,000
2	Nigeria	70	56	59	2,247,000
3	Indonesia	74	67	72	1,150,000
4	Ethiopia	70	65	54	1,134,000
5	Philippines	57	57	57	1,048,000
6	Democratic Republic of the Congo	81	65	55	734,000
7	Brazil	74	68	73	710,000
8	Pakistan	90	83	81	611,000
9	Angola	57	45	36	553,000
10	Myanmar	45	37	44	492,000
11	United Republic of Tanzania	82	81	76	402,000
12	Mozambique	67	61	84	372,000
13	Afghanistan	74	66	63	361,000
14	Somalia	52	42	46	338,000
15	Mexico	83	78	99	317,000
16	Madagascar	65	55	39	304,000
17	Cameroon	76	69		219,000
18	Democratic People’s Republic of Korea	42	41	42	197,000
19	Chad	73	58	55	191,000
20	Viet Nam	87	83	89	187,000

### Appendix A.2. Full Search Strategy

Keywords: “Routine Immunization”[TIAB] OR “Vaccination*”[TIAB] OR “Vaccination Coverage”[TIAB] OR “Childhood Vaccination”[TIAB]

AND

Urban OR Peri-urban OR City OR “Urban center” OR “urban slum”

AND

India OR Nigeria OR Indonesia OR Ethiopia OR Philippines OR “Democratic Republic of the Congo” OR Brazil OR Pakistan OR Angola OR Myanmar OR United Republic of Tanzania OR Mozambique OR Afghanistan OR Somalia OR Mexico OR Madagascar OR Cameroon OR Democratic People’s Republic of Korea OR Chad OR Viet Nam

PubMed

((“Routine Immunization”[Title/Abstract] OR “vaccination*”[Title/Abstract] OR “Vaccination Coverage”[Title/Abstract] OR “Childhood Vaccination”[Title/Abstract]) AND (“Urban”[Title/Abstract] OR “Peri-urban”[Title/Abstract] OR “City”[Title/Abstract] OR “Urban center”[Title/Abstract] OR “urban slum”[Title/Abstract]) AND (“India”[Title/Abstract] OR “Nigeria”[Title/Abstract] OR “Indonesia”[Title/Abstract] OR “Ethiopia”[Title/Abstract] OR “Philippines”[Title/Abstract] OR “Democratic Republic of the Congo”[Title/Abstract] OR “Brazil”[Title/Abstract] OR “Pakistan”[Title/Abstract] OR “Angola”[Title/Abstract] OR “Myanmar”[Title/Abstract] OR “united republic of tanzania”[Title/Abstract] OR “Mozambique”[Title/Abstract] OR “Afghanistan”[Title/Abstract] OR “Somalia”[Title/Abstract] OR “Mexico”[Title/Abstract] OR “Madagascar”[Title/Abstract] OR “Cameroon”[Title/Abstract] OR “democratic people s republic of korea”[Title/Abstract] OR “Chad”[Title/Abstract] OR “viet nam”[Title/Abstract])) AND (2020/3/1:2023/2/3[pdat])

Result = 321

Web of Science

AB = (“Routine Immunization” OR “Vaccination*” OR “Vaccination Coverage” OR “Childhood Vaccination”) AND AB = (India OR Nigeria OR Indonesia OR Ethiopia OR Philippines OR “Democratic Republic of the Congo” OR Brazil OR Pakistan OR Angola OR Myanmar OR United Republic of Tanzania OR Mozambique OR Afghanistan OR Somalia OR Mexico OR Madagascar OR Cameroon OR Democratic People’s Republic of Korea OR Chad OR Viet Nam) AND AB = (Urban OR Peri-urban OR City OR “Urban center” OR “urban slum”) March 2020 to Feb 2023

Result = 287

### Appendix A.3. Summary Table for Search Results

**Title**	**Year**	**Type of Study**	**Location**	**Summary**
Yellow fever vaccination before and during the COVID-19 pandemic in Brazil [14].	2022	Ecological, time series study	Brazil (Nationwide)	A 48.55% reduction in the median number of yellow fever vaccine doses administered in Brazil and in its regions 1 year before the pandemic as compared to 1 year during the pandemic: North (−34.71%), Midwest (−21.72%), South (−63.50%), and Southeast (−34.42%)
Child Vaccination Coverage, Trends and Predictors in Eastern Ethiopia: Implication for Sustainable Development Goals [21].	2021	A population-based longitudinal study	Kersa, Eastern Ethiopia (incl. Harar town)	A little more than a third (39%) of children were fully vaccinated; with highest proportion (45%) seen in 2020 and the lowest (32%) in 2019. Other towns classified as semi-urban had the lowest fully vaccinated proportion even as Harar city saw 45% full vaccination for its children.
Experiences of Urban Slum-Dwelling Women with Maternal and Child Health Services During COVID-19 Pandemic: A Multi-City Qualitative Study From India [15].	2022	A phenomenological study to document MCH experience during COVID-19 pandemic	India: Four states Odisha, Uttarakhand, Chhattisgarh, and Assam. One slum city per state	All participants in this study mentioned that their children were vaccinated during the pandemic with little or no issues. Fear that the child may get infected with COVID-19 was highlighted by caregivers. A few choose a private hospital for child immunization due to fear of COVID-19.
Strategies to revitalize immunization service provision in urban settings of Ethiopia [26].	2021	A qualitative study with a phenomenological study design	Ethiopia: Addis Ababa, Dire Dawa and Mekele	The highlight of the study is that existing immunization service delivery strategies within urban contexts which are mostly fixed sites are not adequate to effectively reach children with vaccines in these settings.
Impact of COVID-19 pandemic on routine immunization of children [16].	2022	Cross-sectional study	Pakistan: Mirpur, Azad Kashmir,	The fear of COVID-19 infection was highlighted as an important factor for delayed vaccination in 65% of respondents.
Disparities in full immunization coverage among urban and rural children aged 12–23 months in southwest Ethiopia: A comparative cross-sectional study [17].	2022	A comparative cross-sectional	Ethiopia: Wolaita zone	Children in urban areas had a higher prevalence of full vaccination than their rural counterparts with a 15.10% (95% CI; 0.102–0.192) point estimate for the difference but still below WHO recommendation. Knowledge and place of delivery were predictor variables.
Effect of intensive training in improving older women’s knowledge and support for infant vaccination in Nigerian urban slums: a before-and-after intervention study [27].	2021	Pre- and post-study	Nigeria: Seven urban slums communities in Ibadan	Participatory learning improved the knowledge about and support for infant vaccination among older women supervising childcare in these urban slum communities.
Assessment of vaccination timeliness and associated factors among children in Toke Kutaye district, central Ethiopia: A Mixed study [22].	2022	A community-based cross-sectional mixed-method study	Ethiopia: Toke Kutaye district, central Ethiopia.	Timeliness of childhood vaccination was 23.9 percent among children aged 12 to 23 months. Urban residence (AOR: 3.15, 95% CI: 1.56–6.4), participation of pregnant women in conferences (AOR: 2.35, 95% CI: 1.2–4.57), institutional delivery (AOR: 2.5)
Parental acceptance of human papillomavirus vaccination for adolescent girls in Lagos, Nigeria [23].	2020	A descriptive cross-sectional survey of adolescent girls’ parents	India: 2 urban and 2 rural schools in Lagos	Tertiary level of education in the mother (cOR = 67.41; 95% CI = 15.25–297.97; *p* = 0.0000), skilled occupation in the mother (cOR = 11.55; 95% CI = 5.55–24.04; *p* = 0.0000), skilled occupation in the father (cOR = 4.10; 95% CI = 2.31–7.28; *p* = 0.0000), are predictors of HPV vaccination.
Second-dose measles vaccination and associated factors among under-five children in urban areas of North Shoa Zone, Central Ethiopia, 2022 [24].	2022	A community-based cross-sectional study	Ethiopia: urban areas of North Shewa Zone, Oromia	With a 90.1% response rate in 372 participants, the coverage of measles second-dose vaccination (MCV2) among children in urban areas was low (42.5%).
Impact of COVID-19 pandemic response on uptake of routine immunizations in Sindh, Pakistan: An analysis of provincial electronic immunization registry data [6].	2020	Quantitative: Secondary data analysis of daily immunization coverage	Pakistan: Sindh urban and rural	The average daily vaccination rate during the COVID-19 lockdown saw a 52.5% decline. Bacille Calmette Guérin (BCG) vaccines saw the highest decline of 40.6% (958/2360). An estimated 8438 children per day missed their vaccines during the lockdown. Areas mostly affected included rural districts, urban sub-districts with large slums, and polio-endemic super high-risk sub-districts.
Impact of the Early Stages of the COVID-19 Pandemic on Coverage of Reproductive, Maternal, and Newborn Health Interventions in Ethiopia: A Natural Experiment [18].	2022	A nationally representative cross-sectional survey	Ethiopia: Addis Ababa	Significant reductions in coverage of BCG vaccination and chlorohexidine use in urban areas were observed in the COVID-19-affected cohort.
Impact and projections of the COVID-19 epidemic on attendance and routine vaccinations at a pediatric referral hospital in Cameroon [19].	2021	A descriptive and retrospective cross-sectional study	Cameroon: Yaoundé	There was a decline in vaccination demand including BCG vaccines, DPT, polio, and MMR in children as well as tetanus vaccines in women of childbearing age, all dropped significantly.
Scared, powerless, insulted and embarrassed: hesitancy towards vaccines among caregivers in Cavite Province, the Philippines [25].	2021	Qualitative: In-depth interviews (IDIs)	Philippines: Cavite Province	Among the reasons for delay or refusal of childhood vaccinations, fear of side effects emerged as the most salient concern, exacerbated by previous negative experiences (including trauma) from a dengue vaccine controversy in 2017.
Missed childhood immunizations during the COVID-19 pandemic in Brazil: Analyses of routine statistics and a national household survey [20].	2021	Qualitative: Ecological, time series study	Brazil: National with subnational statistics	About 20% decline in vaccination rates was seen in children 2 years or older during the months of March and April 2020 during the lockdown in comparison with January and February 2020. The least developed regions of the country were the most affected by missed immunization
